# Risk factors, classification, and operative choices of femur fractures at a Tertiary Hospital: first report from Somalia

**DOI:** 10.1038/s41598-023-39671-9

**Published:** 2023-08-08

**Authors:** Yasin Barkhad Ibrahim, Abdullahi Yusuf Mohamed, Hassan Salad Ibrahim, Abdulkhalek Hassan Mohamed, Hakan Cici, Yahye Garad Mohamed, Nor Abdi Yasin, Hasan May

**Affiliations:** 1 Mogadishu Somalia Turkey Training and Research Hospital, Mogadishu, Somalia; 2 Izmir Democracy University, İzmir, Turkey; 3 Antalya Eğitim ve Araştırma Hastanesi, Antalya, Turkey

**Keywords:** Anatomy, Health care, Risk factors

## Abstract

A traumatic femur fracture is a significant cause of morbidity, affecting one to three million individuals annually. The present is the first study investigated the epidemiological characteristics, risk factors, classification, mechanisms of injury, and early management of femoral fractures in Somalia. This retrospective epidemiological study included all patients with a femur fracture who were admitted for four years between November 2018 and December 2022 to the orthopedic and trauma surgery department. We reviewed patient demographic characteristics, including age and gender, the mechanism of injury, injury characteristics, and the type of fixation performed. We reviewed the radiographs and classified the fracture using the AO/OTA classification system. During the study period, a total of 402 patients were treated for femur fractures; 256 (64%) were males, and 144 (36%) were females. The mean patient age was 47.7 ± 8.5 years. Regarding the anatomical location of femur fractures, the proximal (31A, 31B) was the most common, accounting for 50% of the patients. Femur neck fracture (31B) was the most common in the proximal femur fractures. Gunshot 82 (59.42%) was the leading cause of femur shaft fractures. Most patients with femur shaft fractures were males; 150 (86.20%) and 152 (64.47%) were young patients between 19 and 40 years old. Almost half of the patients (86) with femur shaft fractures had open fractures. The distribution of the mechanism of injury significantly differed according to age (p < 0.001). Younger patients (< 40 years) were predominantly injured due to gunshot injuries compared to elderly cases (> 60 years), where falls from standing height were the primary mechanism of injuries. There was a statistically significant difference between the mechanism of injury and gender categories (p < 0.001). Male patients were injured mainly by gunshots in about 40%, while 80% of fractures in female patients were due to falls from standing height. Female fractures occurred primarily in the proximal, while the males had an equal fracture rate for proximal and shaft fractures. Femur fracture causes significant morbidity and mortality. The study findings revealed that the most common femur fracture type was femur neck fracture, and low-energy injuries were the most common mode of injury in the elderly. Proximal femur fractures occur in older age and mainly in females. Gunshots were the most common cause of femur shaft fractures in Somalia, a country that has struggled with wars for over 30 years.

## Introduction

A traumatic femur fracture is a significant cause of morbidity, affecting one to three million individuals annually^1^. The femur is the most fractured long bone in the body that often necessitates surgical fixation. It is crucial to evaluate the clinical and epidemiological features, mechanisms of injury, and patterns of femoral fractures to determine the suitability of therapeutic approaches and create preventative measures^2^.

When it comes to fractures, epidemiology is generally underestimated^3^. Nowadays, traffic and work accidents have increased due to the active life created by technological developments^4^, while the prevalence of osteoporosis increases with aging^5,6^.

The most common fracture in the elderly is the femoral neck fracture, which can cause substantial disability. The annual number of elderly cases is predicted to climb steadily as the population ages, reaching between 6.3 and 8.2 million patients in two to three decades^6^. In epidemiological studies, subtrochanteric and diaphysis fractures account for 5% to 10% of all fractures. These kinds of fractures are not uncommon in women older than 60 years of age compared to men and increase steeply with age, similar to that hip fractures. Spiral or oblique fractures are the most common radiographically, although transverse fractures have also been documented^7^.

Femoral shaft fractures are a traumatic condition that distorts the entire skeletal system. It can cause death and long-term disability^4^.

Most studies tend to focus on the proximal part due to the frequency and severity of the fracture, especially in an aging population^3^. Nevertheless, Fractures at the distal end of the femur are uncommon, with an incidence of 0.5% of all fractures. Only a few studies have reported incidence rates of distal femur fractures^8^.

There have been no studies regarding femur fractures reported from Somalia. The present study is the first study that examined the prevalence, risk factors, mechanism of injury, anatomical locations, and early management of femur fractures at a tertiary hospital in Somalia.

### Methods

This retrospective epidemiological study included all patients with a femur fracture who were admitted for 4 years between November 2018 and December 2022 to the orthopedic and trauma surgery department of Mogadishu Somalia Turkish Training and Research Hospital. The patient's medical records were respectively reviewed using electronic medical records in the Hospital information system (HIS).

All patients with documented types of femur fracture who were admitted and operated on during the study period, irrespective of age, were included. Patients with incomplete data were excluded from the study.

We reviewed patient demographic characteristics, including age and gender, the mechanism of injury (gunshot, road traffic accident, falls), injury characteristics (open or closed fracture), and type of fixation performed. The international classification of disease, the ninth revision, served basis of medical diagnosis classification.

A group of orthopedic specialists reviewed the radiographs. Each reviewer classifies the fracture using the classification of AO/OTA (Arbeitsgemeinschaft fur Osteosythesefragen/Orthopedic and trauma association)^9^. The details of AO/OTA fracture classification are demonstrated in Table 1.

**Table 1:** AO/OTA fracture classification.

AO/OTA classification	Group
31A Proximal femur fracture trochanteric region	
31A1	Pertrochanteric simple
31A2	Pertrochanteric multifragmentry
31A3	Intertrochanteric
31B proximal femur neck fracture	
31B1	Subcapital
31B2	Transcervical
31B3	Basicervical
32 Femoral shaft fracture	
32A	Simple
32B	Wedge
32C	Complex
33 Distal femur fracture	
33A	Extra-articular fractures
33B	Partial articular
33C	Complete articular

The ethical research board committee of Mogadishu Somalia Turkish Training and Research Hospital (REF. MSTH/9847.) approved the research. In addition, all study participants and a parent of participants under 18 years of age consented to use their medical and surgical data in this study. This study was carried out following the Helsinki Declaration contents.

The data were analyzed using the univariate descriptive study design using the Statistical Package for Social Sciences (SPSS -IBM) for Windows version 26. The frequencies and proportions were presented as point estimates for categorical variables, while the mean (± SD) was employed wherever necessary for quantitative variables. The chi-square test and cross- tabulations were performed to evaluate the association between the variables.

### Ethical approval

The ethical research board committee of Mogadishu Somalia Turkish Training and Research Hospital (REF. MSTH/9847.) approved the research.

### Consent

All study participants and a parent of participants under 18 years of age consented to use their medical and surgical data in this study.

## Results

During the study period, a total of 402 patients were treated for femur fractures; 256 (64%) were males, and 144 (36%) were females. The mean patient age was 47.7 ± 8.5 years.

Regarding the anatomical location of femur fractures, the proximal (31A, 31B) was the most common, accounting for 48.75% (n = 196) of the patients. Falls from standing height or less (low energy trauma) were the primary injury mechanism (Table 2). Femur neck fractures were significantly more common in females than males (55% vs. 45%), while trochanteric fractures had similar rates (47% vs. 52%). More than half of patients with proximal femur fractures were aged between 60 and 79 years, and around a quarter were older than 80.

**Table 2:** Injury characteristics.

AO/OTA	No %	Mechanism of injury				Energy		
		Gunshot	RTA	Fall	Sports injury	Low	Moderate	High
Proximal								
31A	18.91	16	2	58	0	76	0	0
31B	27.36	0	0	110	0	102	0	8
Shaft								
32A	20.90	32	40	10	2	10	2	72
32B	3.48	8	2	2	2	2	2	12
32C	14.93	42	14	2	2	2	2	56
Distal								
33A	6.47	16	2	8	0	8	0	16
33C	2.99	2	2	6	2	6	2	2

According to the AO/OTA classification, femur neck fracture (31B) was the most common in the proximal femur fractures. Its sub-classification, bicervical femur neck fracture (31B3), was the most frequent.

Femur shaft fractures (32A, B, and C) constituted 43.28% (n = 174) of all femur fractures, with which gunshot 82 (59.42%) being the leading cause of fracture flowed by road traffic accidents 56 (40.5%). Most patients with femur shaft fractures were males, 150(86.20%), and most 150(64.47%) were young patients between 19 and 40 years old. Almost half of the patients (86) with femur shaft fractures had open fractures. According to AO OTA classifications, the cases were distributed as follows: 32A for 90 cases (A1 10%, A2 2.5%, A3 10%), 32B for 18 patients (B1, 0.5%, B2 3%, and B3 1%), 32C for 80 patients (C2 1C3 11). The simple type 31A of femur shaft fractures, transverse, and spiral fractures occur in an equal distribution (Table 2).

Distal femur fractures were the least common, accounting for 7.96% (n = 32) of the patients. The primary mechanism of injury was gunshot wounds in 18 patients (52%), and 14 (41.17%) fell from standing height. According to AO/OTA classification, type A was 26 cases, and type C was 10 cases.

There was a statistically significant difference between the mechanism of injury and gender categories (p < 0.001). Male patients were injured mainly by gunshots in about 40%, while 31% by falls and 21% were due to road traffic accidents. In contrast, 80% of fractures in female patients were due to falls from standing height. The distribution of the mechanism of injury significantly differed according to age (p < 0.001). Younger patients (< 40 years) were predominantly injured due to gunshot injuries compared to elderly cases (> 60 years), where falls from standing height were the primary mechanism of injuries (Table 2).

**Figure 1:**
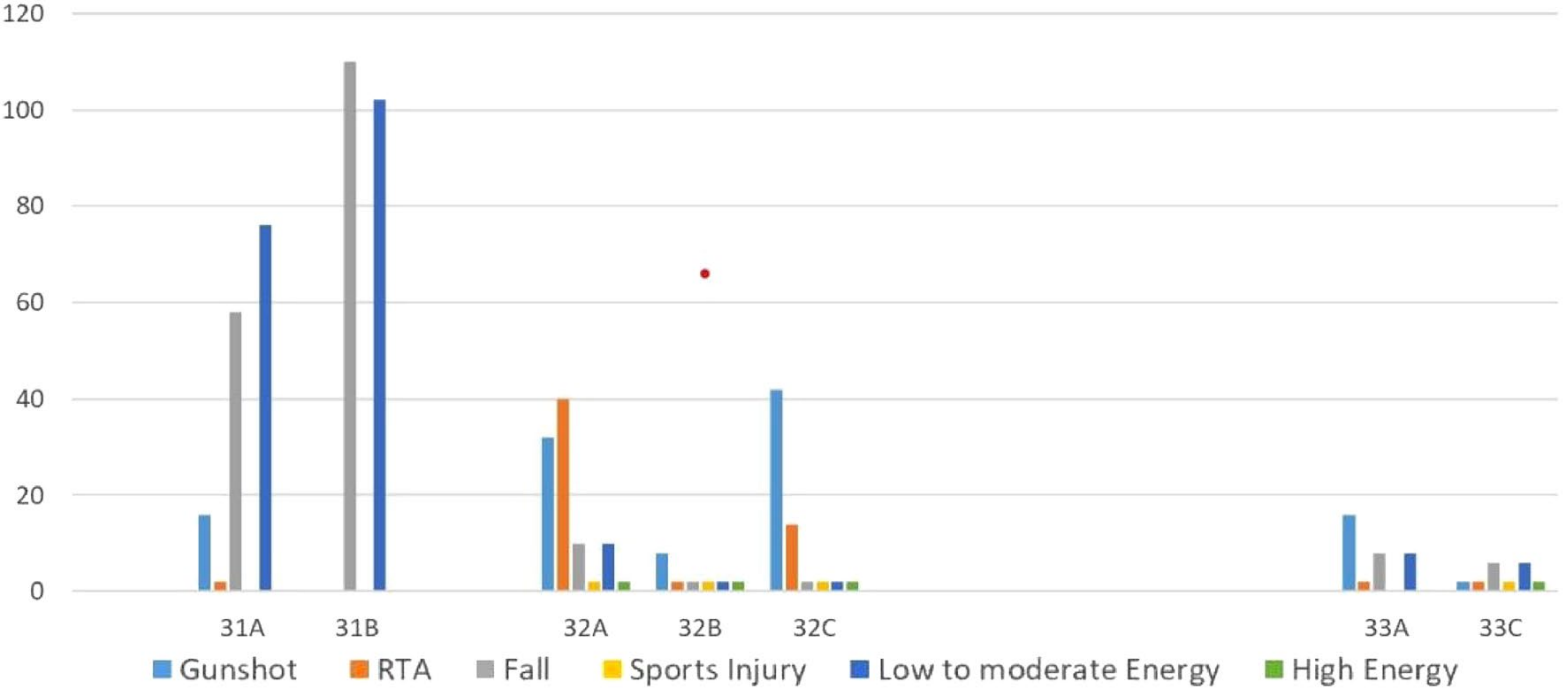
Distribution of mechanism of injury among patients.

Regarding gender distribution of femur fractures, female fractures occurred mainly in the proximal, while the males had an equal fracture rate for proximal and shaft fractures. There was no difference in the distribution of distal femur fracture based on gender (Table 3 and Fig. 1).

**Table 3:** Distribution of femur fractures among age groups and gender.

	Age group						Gender	
	< 18 years	19–39 years	40–59 years	60–79 years	> 80 years		Male	Female
Proximal								
31A	2	16	12	28	16	36		40
31B	2	6	18	64	30	54		66
Shaft								
32A	12	46	14	12	6	72		18
32B	4	8	4	2	0	18		4
32C	4	44	10	4	0	60		2
Distal								
33A	4	6	4	6	4	4		6
33B	0	0	0	4	0	12		4
33C	2	0	2	2	2	6		0

The implant of choice for trochanteric (31A) fractures, both stable and unstable, was a cephalomedulary nail, while femur neck fractures were mainly treated with a prosthesis.

An external fixator construct was used to temporarily fix patients' 17 out of 86 open femur fractures. These patients had hemodynamic instability, huge defect, or contaminated fractures. A significant number of patients with open fractures were directly treated with intramedullary nailing. Ante grade nailing was the treatment of choice for most patients with femur shaft fractures in our study, with the patient in the lateral position without a fracture table (Table4).

**Table 4:** Early management of femur fractures.

Fracture type	Management type	Fixation implant	No. of patients
Proximal			
Intertrochanteric (31A)	Definitive	Proximal femoral nail	76
Femur neck fractures(31B)		Prosthesis	120
Shaft			
Open fractures regardless of AO	Temporary	External fixation construct	30
Closed fractures	Definitive	Intramedullary nail	144
Distal			
Closed fractures	Definitive temporary	Plate ostheosenthesis	28
Open fractures		External fixation construct	6

## Discussion

The present is the first study to evaluate the clinical and epidemiological characteristics, mechanisms of injury, and early management of femoral fractures reported from Somalia, an African horn country of around 15 million populations where these kinds of fractures are common**.**

There is a difference in fracture sites among age groups. In our study, more than half of the patients with proximal femur fractures were aged between 60 and 79 years. A retrospective cross- sectional study by Shao-Chun Wu et al. from Taiwan reported that older patients had a higher likelihood of proximal type A and B fractures. They also reported having a lower risk of sustaining fractures of the femur shaft and distal, which is in line with our study findings^10^. The incidence of proximal femur fractures increases by 2–3 times after 50 years, as reported by Sambrook et al.^6^.

Femur neck fractures were the most common traumatic injuries associated with increasing age, as observed in our study^6^. Femur neck fractures were slightly higher than trochanteric fractures in our cohort. In contrast, in a retrospective study by Tanzi L and his associates, femur neck fractures were approximately equal to peritrochanteric fractures^9^.

Similar to our study findings, Ghouri SI and colleagues reported a preponderance of femur shaft fractures in the male gender (89.4%) and young age group with a mean age of 30 years^2^.

Another study by Burç et al. also showed a male predominance of femur shaft fractures^4^. The above findings also align with a recent study from a tertiary center in Shout East Nigeria due to this age group's highly active lifestyle^11^.

Fractures at the distal end of the femur are uncommon, with only an incidence of 0.5% of all fractures described in the literature^8^. More information about the incidence and epidemiology of distal femur fracture is needed in the literature. Only a few studies have reported incidence rates of distal femur fractures. A retrospective epidemiological study of distal femur fractures reported from Denmark shows a female predominance of distal fractures^8^. A nationwide register study of 417,840 fractures in Sweden across 16 years by N. Lundin et al. evaluated the diverging trends for potentially lethal fractures^12^. The authors reported a female predominance of distal fractures.

In contrast to the above findings, our study shows equal distribution of distal femur fractures in gender categories. This difference may be due to the high incidence of distal femur fractures in males caused by a gunshot in our study. Court-Brown CM, Caesar B. reported an increased risk of distal femur fracture with aging^13^. Our data showed increased distal femur fracture with age.

The mechanism of injury is the causative mechanism that leads to the initiation and growth of cracks and the complete fracture of the bone fracture. These injuries were either high-energy gunshots, road traffic accidents, sports injuries, or low-energy trauma, such as falling from a standing height in a vulnerable aging skeleton^14^.

Minor falls were the leading cause of both femur neck (31B) and trochanteric (31A) fractures in a Nigerian retrospective study^15^, which corresponds to our research. This is related to increased hip stiffness secondary to osteoarthritis, which leads to falls^16^.

A South African study regarding gunshot wounds of the hip reported that most gunshot injuries to the proximal femur were peritrochanteric fractures which also matches our results^17^.

A 4-year study from only a level 1 trauma center in Qatar by Ghouri et al*.* about patterns, management, and outcome of traumatic femur fracture concluded that road traffic accident was the leading cause of femur shaft fracture^2^. Similar findings were also reported in a study of the pattern of femoral fractures and associated injuries in a Nigerian tertiary trauma center^15^. In contrast, our study revealed that gunshots were the most common cause of femur shaft fractures in Somalia, followed by road traffic accidents and falls. This significant difference is mainly due to the war in our country and the great use of weapons in urban areas.

Femur fractures in children are significant trauma with severe complications and disability. Anatomically, it may involve all parts of the femur bone and can be seen in all pediatric age groups^18^. A retrospective epidemiological study of pediatric femoral fractures reported from Switzerland reported that two-thirds of femur fractures in children are caused by falls and RTA, consistent with our results^19^.

According to AO/OTA classification, femur neck fracture (31B) is the most common for the proximal femur fractures, in which its sub-classification, biscervical femur neck fracture (31B3), is the most frequent. The most common type of trochanteric fracture was 31-A1 and 31-A2 in data from the Swedish fracture register by Leif Mattisson and his co-workers, consistent with our cohort^20^.

Type A of extra-articular fracture (33A) was the most common type of distal femur fracture in a population-based epidemiological study reported by R Elsie and colleagues, corresponding to our study^13^. Our analysis also shows more type 33C fractures than type 33B fractures, consistent with the study epidemiology of distal femur fractures from France by pitue and his team^3^.

The pattern of closed femoral fractures is frequently observed due to the femur's soft tissue cover, which contrasts with the tibia fractures. The majority of femur shaft fractures were closed, as reported in a study from Qatar^2^. In contrast, our study shows that almost 50% of femur shaft fractures were open. This significant difference can be explained by the high incidence of femur shaft fractures due to gunshots in our countryside.

This study has certain limitations owing to the retrospective study design and data retrieval from the registry database. This study did not address mortality, the functional outcome of radiological union (nonunion, malunion, extended delayed union) and clinical follow-up details about physical therapy, early mobilization, and counseling. The present study is the first study that examined the prevalence, mechanism of injury, anatomical locations, and early management of femur fractures at a tertiary hospital in Somalia. The study results represent demographic characteristics and management options for surgically treated femur fractures in the country.

## Conclusion

Femur fracture causes significant morbidity and mortality. The study findings revealed that the most common femur fracture type was femur neck fracture, and low-energy injuries were the most common mode of injury in the elderly. Proximal femur fractures occur in older age and mainly in females. Gunshots were the most common cause of femur shaft fractures in Somalia, a country that has struggled with wars for over 30 years. Since our study is being conducted in a single center, more research on the prevalence of national trauma and specialized management of femur fractures is needed to improve healthcare staff expertise and patient care quality. As well as the implementation of a national trauma registry and improvements of decentralized trauma centers throughout the country is necessary to tackle femur-related morbidities.

## Data Availability

The datasets used and/or analyzed during the current study are available from the corresponding author upon reasonable request.
