# Author Correction: Risk factors, classification, and operative choices of femur fractures at a Tertiary Hospital: first report from Somalia

**DOI:** 10.1038/s41598-023-40471-4

**Published:** 2023-08-16

**Authors:** Yasin Barkhad Ibrahim, Abdullahi Yusuf Mohamed, Hassan Salad Ibrahim, Abdulkhalek Hassan Mohamed, Hakan Cici, Yahye Garad Mohamed, Nor Abdi Yasin, Hasan May

**Affiliations:** 1 Mogadishu Somalia Turkey Training and Research Hospital, Mogadishu, Somalia; 2 Izmir Democracy University, İzmir, Turkey; 3 Antalya Eğitim ve Araştırma Hastanesi, Antalya, Turkey

Correction to: *Scientific Reports* https://doi.org/10.1038/s41598-023-39671-9, published online 08 August 2023

The original version of this Article contained an error in the spelling of the author Hakan Cici which was incorrectly given as Hacan Cici.

The original Article has been corrected.

